# “It’s so hard taking pills when you don’t know what they’re for”: a qualitative study of patients’ medicine taking behaviours and conceptualisation of medicines in the context of rheumatoid arthritis

**DOI:** 10.1186/s12913-017-2246-8

**Published:** 2017-04-26

**Authors:** Boitshoko Kobue, Shirra Moch, Jennifer Watermeyer

**Affiliations:** 1 Department of Pharmacy and Pharmacology, School of Therapeutic Sciences, Faculty of Health Sciences, University of the Witwatersrand, 1 Jan Smuts Ave, Braamfontein, Johannesburg, 2050 South Africa; 2 Department of Pharmacy and Pharmacology, School of Therapeutic Sciences, and Centre for Health Science Education, Faculty of Health Sciences, University of the Witwatersrand, 1 Jan Smuts Ave, Braamfontein, Johannesburg, 2050 South Africa; 3 Health Communication Research Unit, School of Human and Community Development, University of the Witwatersrand, 1 Jan Smuts Ave, Braamfontein, Johannesburg, 2050 South Africa

**Keywords:** Adherence, Medicine taking, Conceptualisation of medicine, Rheumatoid arthritis, Chronic illness, Traditional remedies, CAM

## Abstract

**Background:**

Patients with chronic illnesses are often required to take lifelong medication to alleviate symptoms and prevent disease progression. Many patients find it difficult to adhere to prescribed medication for various reasons, some of which may link to the way they conceptualise medicines and understand their illness and treatment. This study explores the medicine taking behaviours of patients presenting with Rheumatoid Arthritis (RA), a chronic inflammatory autoimmune disease. We focused particularly on patients’ conceptualisation and understanding of medicines within this disease context, against a backdrop of scarce healthcare resources.

**Methods:**

We conducted semi-structured interviews with 18 female patients at a rheumatology clinic in South Africa, as well as a review of participants’ medical records. We conducted a secondary analysis of the data using thematic analysis and framework analysis principles.

**Results:**

Participants reported a range of medicine taking behaviours including self-medicating, adding complementary and alternative medicines (CAM) or traditional remedies, and sometimes acquiring prescribed medicines illegally. Participants provided insights into their understanding of what constitutes a medicine and what substances can be added to a prescribed regimen, which impacted on adherence. Importantly, the majority of participants demonstrated poor understanding of their illness, medications, regimens and dosage instructions.

**Conclusions:**

Medicine taking in the context of RA, within the studied demographic, is complex and appears strongly mediated by individual and contextual factors. Poor patient understanding, individual conceptualisation of medicines and medicine taking, and the availability of a range of additional medicines and remedies impact on adherence. Based on these findings, we make some suggestions for how healthcare providers can play a greater role in educating patients living with RA about medicines, CAM and traditional remedies, as well as medicine taking behaviours.

## Background

‘Medication adherence’, or the extent to which a patient takes a medication as prescribed [1], remains an on-going challenge for many patients in various disease contexts. Research has shown that 30–50% of prescribed medications are not taken as recommended [2], and there are numerous reasons why adherence may be difficult for patients to achieve [3]. Poor adherence may lead to failure to reach treatment outcomes, the exacerbation of symptoms and deterioration of health [4].

Adherence can be particularly challenging for patients living with chronic conditions, because they are often required to take medication for an extended period, sometimes for the rest of their lives. Chronic medication taking may necessitate lifestyle changes to accommodate the addition of prescribed medication into daily routines [4]. In most cases, chronic conditions require polypharmacy to achieve desired treatment outcomes, which can be complex and confusing due to the increased pill burden, involvement of different medical specialists and lack of cohesion in medicine instructions [5]. Patients may choose to prioritise one medication over another, and adherence to one medicine does not predict adherence to others [6]. Patients may modify or regulate their medication intake in an attempt to exert control over their condition [7].

‘Non-adherence’ refers to intentional non-adherence (which may involve a choice not to take medications or a lack of persistence with adherence) or non-intentional non-adherence (which may involve incorrect adherence due to, for example, misunderstanding dosage instructions) [4, 8]. Pound et al. describe how patients typically ‘experiment’ with medication regimens, engage in strategic non-adherence behaviours, use medications symptomatically and weigh the pros and cons of taking medications [9]. Clifford et al. [10] describe the necessity-concerns framework, which highlights how patients weigh their perceptions about the need to use a particular medication against their concerns about the medication. Intentional non-adherers may have more concerns about medications and doubt the need for the medication, more so than adherers and non-intentional non-adherers. Adherence is thus dynamic.

The act of taking a medicine involves negotiation of the voice of medicine and the voice of the lifeworld [11, 12] or healthworld [13]. Patients often have particular ideas and expectations of medications that may be mediated by a range of factors including cultural perspectives on illness and treatment, their conceptualisation of what constitutes a medicine, prior experiences of medicine taking, and personal ideas about the necessity and usefulness of medications [9, 14, 15]. Health professionals also have specific ideas about medications and adherence, and they are bound by prescription guidelines and medical knowledge. These perspectives often do not align easily, however, which may explain why some patients ‘experiment’ with regimens or do not adhere to prescribing instructions correctly.

Although adherence to prescribed chronic treatment is a potentially daunting objective, it may be facilitated if patients are equipped with the necessary information to increase understanding of their illness and its treatment [2, 16]. Efficient communication between healthcare providers and patients is essential to ensure effective information giving and understanding of administration instructions, create rapport and trust in the healthcare relationship [17], encourage ‘mutually agreed tailoring’ of medication regimens according to patient needs and experiences, and ultimately promote successful adherence [18, 19].

This study focuses on a group of patients with Rheumatoid Arthritis (RA) living in South Africa.

RA is a chronic inflammatory disease that causes functional impairment, pain and reduced quality of life. It is progressive in nature, so the inflammation, deformity and disability increase over a period of time. Accurate diagnosis and early introduction of disease-modifying medication may induce remission, but this relief can only occur if patients adhere to prescribed treatments. The mainstay of RA treatment is a class of immunomodulatory drugs called Disease Modifying Anti-Rheumatic Drugs (DMARDs). These drugs are often used in conjunction with Non-Steroidal Anti-Inflammatory Drugs (NSAIDs) and corticosteroids, which are used predominantly for symptomatic relief. Treatment regimens can be altered a number of times depending on the disease activity [20].

Should patients fail on DMARD and NSAID therapy, a class of drugs called Biologicals can be introduced. Biologicals may however predispose patients to the risk of reactivation of latent infections such as Tuberculosis (TB) [21]. In a country such as South Africa where both TB and HIV already burden the healthcare system, it is important to limit preventable exacerbation of infections. In the case of RA patients, it becomes essential to ensure that DMARD and NSAID therapy is successful in order to avoid introducing Biologicals. Promoting adherence to prescribed RA medication is one way of achieving this goal.

Adherence in patients with RA is typically low [22]. The progressive nature of RA and frequent treatment alterations may lead patients to turn to herbal or alternative treatments to alleviate pain and joint inflammation [23]. While there may be a number of reasons for poor adherence in this disease context, patient knowledge, understanding and beliefs about the illness and its treatment and poor patient-healthcare provider relationships seem to present strong reasons why some patients do not adhere to treatment regimens [22]. Thus, the role of communication seems central in promoting successful adherence in this disease context.

Although a few studies on treatment and social aspects have been conducted around the world [24–27], RA has not received adequate attention when compared to other conditions, particularly in the context of developing countries like South Africa. Providing RA care in such contexts is challenging as a result of social, economic, systemic and poverty-related factors. The disease is often neglected in favour of more pressing health epidemics such as HIV/AIDS and TB, despite the fact that patients living with RA in these contexts often require additional care for such co-morbidities which further complicates treatment regimens [28].

South Africa provides an interesting context for studying medicine taking and adherence in chronic conditions [12]. The healthcare system faces a distinct set of systemic and socio-economic challenges that can negatively affect patients’ ability to achieve adherence [29–32], including poor healthcare provider-to-patient ratios; medication stock shortages; high levels of poverty and illiteracy amongst patients; language and cultural barriers in the clinic; patients consulting traditional healers while also consulting mainstream medical doctors; and the availability of a wide range of complementary and alternative medicines (CAM).

This study forms part of a larger research project that described factors influencing adherence behaviours amongst a group of patients living with RA, including the impact of the illness on the lifeworld of the patient and patients’ experiences of healthcare services [33]. In this paper, we focus particularly on how participants conceptualise medicines, their understanding of treatment regimens, and how these factors reportedly affect their medicine taking behaviours.

## Methods

We chose a qualitative approach for the study as it gave patients the opportunity to express their medicine taking behaviours in their own words. It also allowed insight into patient experiences of their diagnosis and prescribed medications.

The study was conducted at an outpatient rheumatology clinic at a tertiary hospital in Gauteng, situated in a township area characterised by socio-economic and cultural diversity, a history of poverty and poor infrastructure. Patients experiencing symptoms of RA – for example, joint pain – first attended their local clinics or primary healthcare facilities where they were treated with analgesics, anti-inflammatory agents and oral corticosteroids. If symptoms persisted, intra-articular corticosteroids were administered alongside oral agents. If treatment failed again and the doctor suspected an inflammatory arthritis, the patient was referred to the rheumatology clinic at the hospital. In addition to individual consultations, patients attended group counselling sessions at the RA clinic where issues related to living with RA were discussed and they could ask questions about their illness and treatment.

Ethical clearance was obtained through the Human Research Ethics Committee (Medical) of the University of the Witwatersrand. Participants were invited to participate and were provided with written and verbal information about the study. Written informed consent was obtained from all participants, including consent to publish demographic information.

Data collection primarily involved semi-structured interviews with 18 female patients (see Table 1) conducted by the first author in participants’ home languages. Although RA also affects males, in lesser proportion to females [34], no male patients attended the clinic during the data collection period. As a result, males were not included in the investigation, and this is acknowledged as a limitation of the study.

**Table 1:** Patient demographics

Code	Age (yrs)	Gender	Diagnoses^a^	Home language	Education level	Employment status
P1	54	F	RA	isiZulu	Grade 10	Fruit & Veg packer
P2	69	F	RA, DM, HT	isiXhosa	Grade 4	Unemployed
P3	73	F	RA, HT	Sesotho	Grade 6	Pensioner
P4	40	F	RA	Setswana	Tertiary	Unemployed
P5	57	F	RA, HT, Ch	English	Grade 12	Unemployed
P6	66	F	RA, DM, HT	Setswana	Grade 9	Pensioner
P7	44	F	RA, HT	isiZulu	Grade 12	Unemployed
P8	64	F	RA	isiZulu	Grade 8	Pensioner
P9	23	F	RA	Sesotho	Grade 12	Waitress
P10	60	F	RA, HT	Xitsonga	Grade 8	Unemployed
P11	27	F	RA	isiXhosa	Tertiary	Admin clerk
P12	76	F	RA, HT	Setswana	Grade 10	Pensioner
P13	59	F	RA, HT	Setswana	Grade 10	Unemployed
P14	46	F	RA	Sesotho	Grade 12	Unemployed
P15	57	F	RA, DM, HT, Ch	Sesotho	Grade 10	Unemployed
P16	59	F	RA, EP, HT, Allergy	Setswana	Grade 8	Pensioner
P17	49	F	RA, HIV	Sepedi	Grade 11	Unemployed
P18	48	F	RA	English	Grade 11	Labeller

^a^ *HT* Hypertension, *DM* Type 2 Diabetes Mellitus, *EP* Epilepsy, *Ch* Hyperlipidaemia, *HIV* human immunodeficiency virus infection

We used purposive sampling for this study. The participants were individually approached, at random, in the waiting room before their scheduled appointments with the rheumatologists at the clinic. Between three and five interviews were conducted on a weekly basis, over the course of 5 weeks, averaging 30 min an interview. The interviews were video recorded for the purpose of capturing discussions in which medicines were used as props. Participants were asked about their treatment seeking journey, medication taking behaviours and the factors that affect how and when the medication was taken, their understanding of their illness and the manner in which the prescribed medication contributed towards managing and alleviating their symptoms, and their incorporation of the regimen into daily routines. Data saturation was reached after 15 interviews and the additional three interviews were conducted to verify the themes obtained. A review of participants’ medical records was also conducted, which enabled a comparison of their interview responses with the doctors’ notes and pharmacy records.

The interviews were transcribed and translated into English by the first author. To ensure reliability, a random sample of five transcripts was back translated by an independent translator.

We used thematic analysis [35] to conduct a secondary, supplementary analysis [36] of the data and identify and categorise themes and subthemes related to participants’ medicine taking behaviours and understanding of the treatment regimen. Although our initial analysis focused on adherence and medicine taking behaviours [33], this supplementary analysis enabled us to examine participants’ conceptualisation of medicines, medicine taking behaviours and understanding of medication regimens in more depth. The analytic process was moderated by a series of peer debriefing sessions between all authors that served to further limit researcher bias and ensure the accuracy of reported results. We conducted line-by-line coding of each interview and then identified and defined themes using a framework-type approach [37]. We compared the responses provided by participants when asked about their medication regimens to the information noted by doctors and pharmacists in their medical record about prescriptions and treatment regimens.

## Results

### Patient demographics

The mean age of the 18 female participants in this study was 53.9 years (SD = 14.4) and the range was 23–76 years. Of the group, 9 participants were unemployed, 5 were pensioners and only 4 participants were currently working. Eleven patients had concomitant illnesses, the most common of which was hypertension (*n* = 10), followed by type-2 diabetes mellitus (*n* = 3).

**Fig. 1:**
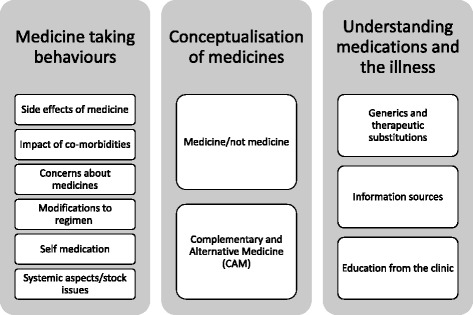
Summary of themes deduced from abstraction of data concerning medicine-taking behaviours, conceptualisation of medicines and understanding of medication and illnesses

In this analysis, the predominant themes included medicine-taking behaviours, conceptualisation of medication and understanding of medication and illnesses. Sub-themes are discussed in the sections below, and summarised in Fig. 1.

### Medicine taking behaviours

There is a wide array of issues that contribute to whether patients will take a prescribed medicine or not and whether they will take it correctly. The themes identified in this data encompass personal, systemic, cultural and socio-economic factors and seem to link strongly to patients’ conceptualisation of medicines and their understanding of their illness and treatment.

All participants initially reported that they took their prescribed medication regularly. They described how the medication had helped to alleviate the symptoms of RA and reduce the pain experienced prior to diagnosis. It became apparent however upon further discussion that ‘regularly’ did not necessarily mean that medication was taken daily as prescribed. As evidenced in Table 2, participants reported a range of medicine taking behaviours, including abstaining or ‘taking a break’ from all or certain medications; supplementing regimens with CAM or traditional remedies; and self-medicating with over-the-counter (OTC) medications or other drugs:

**Table 2:** Patient reports of modifications to treatment regimens and understanding of illness and treatment

	Reported modifications to treatment regimen	Understanding of treatment regimen (how drugs work and/or how to take them) and correlation with medical records	Understanding of illness (es) (incl. disease progression, symptomatology)	Adherence classification
P1	Self-medicates despite being told not to do so by the doctor; takes an OTC analgesic	Poor understanding of how drugs work	Limited understanding of illness. Cartilage is being eaten away, causing joint pain. RA is caused by many years of performing household chores that involve use of water	Intentional non-adherer
P2	Does not use traditional medicines or CAM; self-medicates with previously prescribed pain medicines; increases insulin when sugar levels are too high	Poor understanding of regimen; discrepancies in records versus patient description of drugs	Cannot link her symptoms to her various chronic conditions Hard work/chores cause RA	Intentional non-adherer
P3	Scared of mixing medicines	Does not know what drugs are for, just takes them	Limited understanding of illness RA is caused by over-exposure to cold temperatures	Adherer
P4	Took ‘bone medicine’ acquired illegally from hospital; deliberately skips evening doses when not in pain	Basic understanding of what drugs are for, does not understand how they work	Limited understanding of illness Fluid in the joints has run out, causing pain when cartilage gets worn out. No known cause of RA, you just get it	Intentional non-adherer
P5	Self-medicates with topical mustard and garlic oil; drinks lemon water with honey	Did not include all drugs in description of regimen; does not understand how drugs work	Limited understanding of illness Red blood cells are fighting the white blood cells. RA might be genetic	Non-intentional non-adherer
P6	Sometimes feels like taking a break from medicines; scared to mix medicines	Takes so many pills, she forgets the names; clear understanding of how to take drugs, which does not match records; good understanding of RA and DM drugs	Understands RA and DM best, although vaguely; poor understanding of HT and Ch Bones are being eaten away. RA caused by over-working yourself.	Intentional non-adherer
P7	Drinks ‘Forever Living’ (a tonic containing aloe vera extract)	Poor understanding of HT drugs; knowledgeable about RA drugs	Not concerned about HT; poor knowledge of HT, good knowledge of RA Immune system is attacking the joints and causing inflammation; cartilage gets eaten away. RA might be hereditary but doctors are uncertain.	Intentional non-adherer
P8	Does not take chloroquine because it ruins her eyes; drinks ‘Lavida tea’ (an organic herbal tea)	Unable to recall all drugs; not taking everything correctly; uncertain about how drugs work	Limited understanding of illness Nature of disease not explained. RA just happens; not related to hard work or chores.	Intentional non-adherer
P9	Does not self-medicate since proper diagnosis was made	Not taking drugs as prescribed; taking drugs that were previously prescribed; does not know names of drugs; does not know what drugs are for	Does not understand illness The gel between the bones is finished causing pain when they rub together. Cause is unknown, not chores related.	Intentional non-adherer
P10	Does not self-medicate	Could not recall all drugs but seems to be taking them correctly; taking triple dosage in error for one drug; does not understand what drugs are for	Limited understanding of illness Exposure to water and cold causes RA	Non-intentional non-adherer
P11	Does not self-medicate or mix medicines due to possible side effects	Good understanding of drugs	Good understanding of illness Immune system incorrectly identified something in the body as foreign and has mounted an attack. Cartilage between bones has been eaten away, causing pain. Cause unknown	Adherer
P12	Does not self-medicate because she’s afraid of side effects	Describes drug regimen correctly, but does not know which drug is for which condition	Less concerned about HT than RA Cartilage has been eaten way causing pain. No cause	Adherer
P13	Initially self-medicated with NSAIDS; uses aloe (traditional medicine); stops taking medicines sometimes, just to see how her body will feel	Could not recall all drugs but seems to know how to take them correctly	Limited understanding of illness Bones and joints swell and cause pain. Cause is still unknown	Non-intentional non-adherer
P14	Understands importance of calcium but does not take it due to taste and side effects; self-medicates with joint support pills and pain killers obtained illegally from pharmacy	Understands drugs for each condition; mostly clear on how to take drugs and how they work	Limited understanding of illness RA caused by hard work and exposure to cold. Hereditary	Intentional non-adherer
P15	Overdoses on medicines to help her sleep; sometimes skips HT medicine; self-medicates with ‘phila’ (traditional remedy) for DM	Could not remember all drugs; some understanding of what they are for; lack of understanding leads to overdosing	Does not fully understand all her conditions Bones being eaten away by excess acid. RA caused by the cold	Non-intentional non-adherer
P16	If she misses a weekly dose, she waits until the next week as she was told not to switch days	Some misconceptions about why she takes certain drugs; recognises drug names and knows how to take them	Limited understanding of illness Bones are rubbing up against each other which causes pain because cartilage is worn out. No real cause; could be a curse	Adherer
P17	Does not self-medicate	Seems clear on how to take drugs and what they are for	Limited understanding of illness Fluid between the bones in finished, causing friction between the bones. No cause	Adherer
P18	Was taking OTC medications until they could no longer control pain	Understands how to take drugs and has some understanding of what they are for	Limited understanding of illness The joints are being attacked which depletes the gel between them. Causes friction and pain. Might be a result of smoking but the cause is not known	Adherer

P6: “Sometimes you just want a break from them. You don’t want any pills that day…You just tell yourself that you’re not taking them that day and you’ll just see what happens.”

P2: “Oh, I forgot to tell you about the green pills, Pynstop [an analgesic]. That’s the one I ask for at the chemist.”

‘Self-medicating’ involves the use of non-prescription drugs, often without consultation with a medical practitioner or under their supervision. Importantly, the addition of drugs, CAM or traditional remedies may interfere with the functioning of a prescribed regimen and possibly jeopardise the patient’s health [38]. One participant described her experience of an adverse reaction to taking medication that had not been prescribed by her doctors:

P4: “The problem is when you take medication that hasn’t been prescribed for you, they hurt you here [points to epigastric region]…They burn you here. It feels like you have an ulcer.”

Five of the participants reported that they did not use other medications or remedies and three participants reported that they feared the effects of mixing medicines (presumably referring to traditional remedies and/or CAM). In the extract below, P12 describes her reluctance to use other medications as she is afraid of having to report the repercussions to the doctor:

P12: “I’m afraid of the after effects because if they have after effects, who will I consult?”

Some participants indicated concerns about the medications and the long-term effects on their overall health. P7’s concerns seemed to correlate with findings in other studies of patients reporting fear of dependence of medication, and needing medication to attain a sense of normality [7]:

P7: “It’s helped but at the same time I worry. I’m worried about what it’s doing to me. I don’t want to lie, it bothers me. I’m always on them. They have become my life. I ask myself because our bodies were made to fight for themselves. So now, what’s going on inside mine?”

Participants diagnosed with co-morbidities seemed most likely to alter their prescribed regimens. They tended to prioritise RA over other co-morbid conditions, were often confused about how to take their medications and seemed inclined to self-medicate. These patients often tried to minimise the quantity of medication they took every day in an attempt to reduce the pill burden. As a result, they tended to place higher priority on medications for conditions where symptoms more obviously affected their daily lives, most often prioritising RA because of the associated pain and inability to carry out daily tasks. ‘Asymptomatic’ conditions such as hypertension seemed to be less of a priority and as a result, these medications were not always taken as prescribed:

P7: “In all honesty, I’m not as concerned about the high blood ones. I take them because they’re my pills but it’s not something that’s on my mind. What’s on my mind is this [RA].”

### Conceptualisation of medicines

The idea of what constitutes a medicine was a prominent theme in the data. The term ‘medication’ relates to the pharmacological agent itself, as well as the use and application of the substance [39]. By this definition, not only is the activity of the substance important, but so too is the route of administration. Patients however may not conceptualise medicine in the same way as healthcare providers, especially when adding CAM, traditional remedies or additional drugs to prescribed regimens. Furthermore, factors such as size, texture and taste may impact on patients’ willingness to adhere to medication regimens [40].

In the example below, P1 has been prescribed naproxen, a NSAID:

Interviewer: “Are there times when you take two [naproxen tablets] instead of one, because the pain is that intense?”

P1: “No, I’ve never taken such chances. Instead, I try to find a Panado [OTC analgesic] and I take the Panado because I’m scared of taking two [naproxen tablets] because maybe it’s wrong…I just take a Panado when the pain gets too bad.”

Although P1shows concern about altering the naproxen dose prescribed by the rheumatologist, she does not appear to have concerns about altering the dose of Panado, an OTC analgesic and antipyretic product containing paracetamol. In this case, it appears that she may consider paracetamol as “less of a medicine” or less harmful than the rest of her prescribed treatment, confirmed by her unwillingness to “take chances” with naproxen, while being comfortable with taking additional Panados. Bennin and Rother’s study suggests similar overuse and misunderstanding of OTC painkillers [41].

Several participants reported using CAM:

P5: “I use the mustard and garlic oil for rubbing. Just for rubbing. I don’t drink things.”

P7: “I drink Forever Living…people sell it. There are representatives who sell it. I drink the one for arthritis, the white one. There’s also a brown one and a maroon one…I drink 3 [bottle lids] full in the morning.”

P8: “I drink Lavida teas…There are people who sell them. So they advertise, then invite neighbours over and then make green tea and you drink it while they explain and chat to you.”

When asked if she used any medication in addition to what was prescribed by the doctors, P5 reported that she did not. In the extract above, she makes a distinction between remedies for rubbing and substances that are ingested, and emphatically points out that she doesn’t ‘drink things’. The meaning of ‘things’ is unclear in this instance but having a different route of administration seems to be her main justification for considering it safe to use a CAM product to alleviate her symptoms.

Several participants described the use of medication obtained illegally from a hospital or pharmacy:

P4: “I once took medication that I was told was for bones, given to me by my mother. She got them from my cousin who works at a hospital.”

Although perhaps propelled by drug shortages in the healthcare system, this example seems to reveal the participant’s apparent belief in the legitimacy and potency of ‘medicine’ that comes from a hospital, even though she is not sure what the medicine is for and the process of medical consultation and prescription has not been followed.

### Understanding medications and the illness

Patient understanding of diagnoses and prescribed medications has the potential to affect whether they take their medication and how they take it. The majority of patients did not appear to completely understand their medication and its intended use, as evidenced in Table 2. When asked to describe their drug regimens, participants demonstrated limited understanding of how the medications work; mismatches between the prescription records and patient reports; incorrect demonstration and poor recall of dosage instructions; and limited recall of medication names. In some instances, participants spoke confidently about their medication regimens and how they take each medicine, but this information did not correlate with what was noted in their file by a doctor or pharmacist. We present some examples of misunderstandings below.

Although all participants experienced symptomatic relief from the medications, it was apparent that some might not have understood the preventative nature of chronic medication for RA and the need for strict adherence even though the symptoms appear to be alleviated:

P13: “Sometimes, though, I stop. Maybe for a day, I stop just to see how I will cope…I just want to know how my body would feel…Then it hurts and I sit. At night I think about taking them but I decide against it, just for a day.”

P15: “I skip days with the high blood ones. I don’t want to lie…I want to feel if the dizziness that I get is there when I don’t take it.”

Both the above participants displayed a phenomenon described by Elliott et al. [6] where patients evaluate their medications in an attempt to determine their necessity. The participants appear to display intentional non-adherence in an attempt to exert control over their medication and chronic conditions. In both instances, however, the recurrence of symptoms led to the participants taking the prescribed medication again.

A few participants admitted that they purchase medication that their doctor had withdrawn from their prescribed regimen:

Interviewer: “How do you buy [prednisone] without a prescription?”

P14: “There are [pharmacists] that will sell them to you.”

Prednisone, a corticosteroid, was the drug most participants reported purchasing illegally (without the required valid prescription) as it was said to provide the most pronounced pain relief. These participants did not seem to realise the adverse long-term consequences of this drug, the changeable nature of RA treatment, and the reason why the drug was withdrawn from their regimen.

Generic substitution is standard practice within the hospital pharmacy due to the tender purchasing system and the national drug policy. It was evident however that the idea was not well understood by participants and may not have been adequately explained by healthcare providers. When unfamiliar products were dispensed, adherence was often negatively affected, as evidenced in the following extract about generics:

P8: “Me, when I don’t understand what something is for, I come back with them. Then I ask the doctor what they’re for during my [next consultation]…They [at the pharmacy] shove your things at you. So then I put them aside. If I remember, when I come back to the clinic or to collect my repeat, I bring them with [me] to ask what they’re for.”

Generic substitutions also resulted in confusion around dosages. P10 was prescribed 12,5 mg of an antihypertensive preparation, but was taking double this dose and complained of nocturia. It seemed the pharmacy had dispensed the 12,5 mg dosage as well as a generic 25 mg dosage:

P10: “I take half [of the 25 mg] in the morning and the [12.5 mg] at night.”

Frequent drug shortages also give rise to the practice of ‘therapeutic substitution’ where a chemically different drug with a similar pharmacological scope of activity is dispensed as part of a regular prescription. Patients confuse this practice with generic substitution, which impacts on their understanding and adherence. P14 describes her experience of drugs dispensed as alternatives for each other, but the drugs she mentions are not in fact always equivalent. She understands the need for generic substitution but has misunderstood which drugs are generics. She was given another diclofenac containing product, as a generic to Voltaren which she usually received. On another occasion, she was given naproxen, which belongs to the same class of drugs, when diclofenac containing products were unavailable. She then also mentions a time when prednisone was dispensed in place of her NSAID-containing medication. This is neither a generic nor a therapeutic alternative, therefore, should not have been dispensed to her as such:

P14: “Diclofenac works like Voltaren…Sometimes when you don’t have diclofenac, then there’s naproxen…Sometimes at the pharmacy they don’t give them all to you…Sometimes its naproxen or prednisone. Then they don’t give you diclofenac.”

When asked to explain RA, the majority of participants showed poor understanding of the disease. Explanations generally related to doing too many chores or being exposed to cold temperatures. A few participants had some understanding of the disease related to fluid in the joints or to an immune response. Participants also showed in general poor understanding of the relationship between co-morbidities and good understanding of some illnesses and not others.

P1: “The way I see it, I think because I spent many years working as a domestic worker, doing laundry and ironing. Maybe that’s when it started and I ignored it. I also think using water often, and electricity with ironing, caused it.”

Half the participants mentioned sources of information about RA and its treatment, the most important being the information provided by the clinic. P7 was one of two participants who reported actively seeking information from sources other than the healthcare providers. She also appeared to be one of the best informed of the participants as she was able to correctly describe how the majority of her medication worked, and the side effects that can be attributed to specific medicines. She was also able to explain the involvement of her immune system in the damage to her joints:

P7: “I attend workshops in [a nearby city]. I read books/pamphlets. I want to know what it’s all about. Even when I’m with the doctor, I ask questions about what’s happening to me. What kind of disease is this that doesn’t get better?”

Several participants mentioned that they did not feel they had received adequate counselling and education about their prescribed medication and the healthcare providers were too busy to speak to them so they often leave the clinic with insufficient understanding – again, an issue related to systemic factors and limited resources. Some were aware of their lack of understanding:

P8: “Who will I ask because the doctors are always busy? Who will I ask?”

P10: “It’s so hard taking pills when you don’t know what they’re for…I was doubtful of taking them…but I thought that because they gave them to me at the hospital, its best I took them.”

P8: “Don’t tell me about the pharmacy! If the doctor prescribes something new or changes your routine, at the pharmacy when they give you your medication, if you ask what are those for, some will explain but some don’t even bother. They just shove your stuff at you and put it in your bag.”

## Discussion

Medication taking is a complex phenomenon specific to each patient and is affected by a number of factors including personal, social, systemic, resource and disease factors. Importantly, adherence in the context of RA seems to relate to how patients understand their illness (es) and medication regimen as well as how they conceptualise medicines and medicine taking.

Although conclusions could not be drawn based on the impact of age or level of education on adherence levels across the chosen demographic, the majority of participants in our study demonstrated poor understanding of prescribed medications and of RA itself, as described in Table 2, which appeared to impact on their medicine taking. In some instances, it appears that poor understanding, the complexity of RA medication regimens, the availability of a range of other options (OTC medications, CAM and traditional remedies) and systemic challenges in the healthcare system led participants to make decisions that adversely affected their medication taking habits. The influence of co-morbidities and the burden of managing multiple medication regimens seems to pose particular challenges, as highlighted in studies of other chronic conditions [6, 10, 42, 43]. Patients may believe they are adhering correctly but are in fact not doing so.

Those participants with co-morbidities seemed to prioritise RA over their other chronic conditions, confirming results from previous research [6]. Some participants explicitly stated that they were more concerned about RA than other conditions and this was evident in participants’ relative lack of understanding of all co-morbidities and related prescribed medication regimens. This behaviour was similar to that found in a study by Rifkin et al., where patients, particularly those with complex regimens, tended to prioritise their medication based on their perceived importance of the disease and effects of the medication on their quality of life [44].

Importantly, the lack of understanding amongst this group of participants was present despite the provision of patient education at the RA clinic. Poor patient understanding may arise for a number of reasons, including the frequent mismatch in this context between the language of the doctor and the language of the patient, limited opportunities and time for talking about the illness and treatment, patients not feeling able to ask for clarification of information given, or limited verification of patient understanding [12, 17]. In addition, it seemed that although patient education was provided by the clinic, information giving about medications and illness was not always consistently provided by all healthcare providers in the care team.

Instead of looking at outcomes of the medicine taking process merely in terms of whether or not patients take medicines and follow prescriptions, the results of this study suggest that patients living with RA may take some, but not all, prescribed medications correctly and the latter seems to occur particularly when co-morbidities are present; patients may be passive accepters of the medication regimen and believe they are taking it correctly despite failing to understand the dosage instructions and thus not taking the medicine correctly; and patients may modify the regimen and/or add OTC medications, CAM or traditional remedies.

Adherence cannot purely be looked at from the healthcare provider’s perspective, and our results confirm this notion. The lived experiences, needs and concerns of patients need to be considered in efforts to promote adherence [6]. As described by Leventhal et al. [45] in their self-regulation model, patients’ understanding of their illness and treatment is informed by their experiences of treatment, the healthcare system and also of adapting to their illness (es). Patients’ beliefs about the need for medications as well as their concerns about medications will also impact on their adherence behaviours [10].

Many of the participants in our study appeared to be autonomous patients who did not passively accept the instructions given by healthcare providers. Some took active steps to obtain medications (sometimes via illegal means), self-medicate or ‘enhance’ their regimens with non-prescribed medicines, and seek out information about their illnesses. These actions are likely a direct result of the progressive, debilitating nature of RA symptoms and also factors such as drug shortages at the hospital. In addition, the social context of CAM, which is removed from medicine, is perhaps more accessible to patients and aligns more strongly with their own perceptions of illness and treatment rather than with the medical model [13].

Previous research has emphasized the importance of ensuring that the patient’s right to agency and self-management is considered [46] and their expertise acknowledged [47] by the team of healthcare providers. In order to achieve these goals and empower patients living with RA to make decisions about their health and medicine taking that will ultimately have positive health outcomes, it is essential that they are provided with accurate information about their illness and medication regimen and that they understand this information.

Although not synonymous with adherence, the concept of *concordance* focuses on the interaction between patient and healthcare provider in the context of medicine taking. In particular, concordance embraces the idea that patients have a right to make decisions about whether or not to take medicines [2, 48, 49]. In order for concordance to be achieved, there needs to be better communication between healthcare providers and patients to facilitate improved information giving and patient understanding.

This study underlines the need for healthcare providers to engage in more effective patient education about drug regimens and medicine taking, particularly around topics such as: the dangers of misusing OTC medicines; the advantages and disadvantages of CAM and traditional remedies when taking prescribed medications; generic substitutions; how medicines for different co-morbidities work together; where patients can access additional, credible health related information. Lempp et al. discuss the need fora multidisciplinary approach to deliver patient centered care [50]. Patients require health education, psychological support and advice on lifestyle changes that need to be implemented to achieve desired treatment outcomes. This approach requires access to a number of resources within the healthcare system as well as the availability of trained professionals. In a resource scarce environment like South Africa, it is not always possible to provide this level of quality healthcare to patients.

Although systemic deficiencies within health systems may be difficult to alleviate, some of the issues identified in this study that negatively affect patient adherence may be addressed through improved information giving and patient understanding. Consultation time needs to be utilised to the full and strategies such as eliciting a demonstration of patient understanding may assist in enhancing service efficiency and ensuring patient understanding of drug information [17, 51].

Importantly, communication should embrace a patient-centred, culturally safe approach that acknowledges diverse perspectives about medicines and enables opportunities for talking about traditional remedies and CAM that may be available to patients [12]. As Koenig et al. [52] point out there are ways in which providers can promote communication about CAM and traditional remedies with patients, without necessarily endorsing the use of such products. Healthcare providers should be well informed about the kinds of products that patients may be accessing so that they can counsel patients regarding the concurrent use of these medicines with prescribed regimens and empower patients to make appropriate decisions in this regard. These principles are not unique to the South African context: given the increase in culturally diverse immigrant and refugee populations in many settings, patients may increasingly access traditional remedies and CAM in place of or in conjunction with biomedical treatments.

## Conclusions

The findings of this study emphasise the complex network of issues that affect patients’ medication taking behaviour in the context of RA. Importantly, patient understanding of drug regimens and illness seems to play a particularly important role in determining medicine taking behaviours, as does patients’ conceptualisation of medicines. This study highlights the benefits of qualitative research in exploring the patient’s perspective on medicine taking. Future research avenues should include a focus on medicine taking behaviours in patients with co-morbidities. In addition, given the limitations of secondary analysis, future research should involve asking patients directly about their conceptualisation of medicines.

## Abbreviations

AIDS: Acquired immune deficiency syndrome
CAM: Complementary and alternative medicines
Ch: Hyperlipidaemia
DM: Type 2 diabetes mellitus
DMARDs: Disease modifying anti-rheumatic drugs
EP: Epilepsy
HIV: Human immunodeficiency virus
HT: Hypertension
NSAIDs: Non-steroidal anti-inflammatory drugs
RA: Rheumatoid arthritis
TB: Tuberculosis
